# Neurofunctional and behavioural measures associated with fMRI-neurofeedback learning in adolescents with Attention-Deficit/Hyperactivity Disorder

**DOI:** 10.1016/j.nicl.2020.102291

**Published:** 2020-05-26

**Authors:** Sheut-Ling Lam, Marion Criaud, Analucia Alegria, Gareth J. Barker, Vincent Giampietro, Katya Rubia

**Affiliations:** a Department of Child and Adolescent Psychiatry, Institute of Psychiatry, Psychology & Neuroscience, King’s College London, London, UK; b Department of Neuroimaging, Institute of Psychiatry, Psychology & Neuroscience, King’s College London, London, UK

**Keywords:** ADHD, fMRI, fMRI-neurofeedback, Neurofeedback, Stop task, predictors

## Abstract

Functional Magnetic Resonance Imaging Neurofeedback (fMRI-NF) targeting brain areas/networks shown to be dysfunctional by previous fMRI research is a promising novel neurotherapy for ADHD. Our pioneering study in 31 adolescents with ADHD showed that fMRI-NF of the right inferior frontal cortex (rIFC) and of the left parahippocampal gyrus (lPHG) was associated with clinical improvements. Previous studies using electro-encephalography-NF have shown, however, that not all ADHD patients learn to self-regulate, and the predictors of fMRI-NF self-regulation learning are not presently known. The aim of the current study was therefore to elucidate the potential predictors of fMRI-NF learning by investigating the relationship between fMRI-NF learning and baseline inhibitory brain function during an fMRI stop task, along with clinical and cognitive measures. fMRI-NF learning capacity was calculated for each participant by correlating the number of completed fMRI-NF runs with brain activation in their respective target regions from each run (rIFC or lPHG); higher correlation values were taken as a marker of better (linear) fMRI-NF learning. Linear correlations were then conducted between baseline measures and the participants’ capacity for fMRI-NF learning. Better fMRI-NF learning was related to increased activation in left inferior fronto-striatal regions during the fMRI stop task. Poorer self-regulation during fMRI-NF training was associated with enhanced activation in posterior temporo-occipital and cerebellar regions. Cognitive and clinical measures were not associated with general fMRI-NF learning across all participants. A categorical analysis showed that 48% of adolescents with ADHD successfully learned fMRI-NF and this was also not associated with any baseline clinical or cognitive measures except that faster processing speed during inhibition and attention tasks predicted learning. Taken together, the findings suggest that imaging data are more predictive of fMRI-NF self-regulation skills in ADHD than behavioural data. Stronger baseline activation in fronto-striatal cognitive control regions predicts better fMRI-NF learning in ADHD.

## Highlights

• Test between baseline measures and generic fMRI-Neurofeedback learning in ADHD.
• Left fronto-striatal fMRI activation during inhibition predicted fMRI-NF learning.
• Activation in posterior regions was associated with poor fMRI-NF learning.
• Clinical or cognitive measures did not predict fMRI-NF learning.
• Brain function predicts NF learning in ADHD better than behavioural measures.

## Graphical abstract

**Figure:**
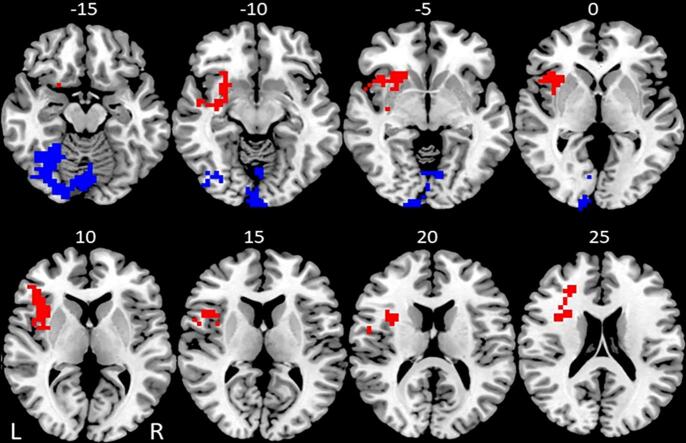

## 1 Introduction

Attention-Deficit/Hyperactivity Disorder (ADHD) is characterised by age-inappropriate, persistent and impairing symptoms of inattention and/or hyperactivity/impulsivity (American Psychiatric Association APA, 2013). ADHD is the most common neurodevelopmental disorder, with a worldwide prevalence of about 7% in children (Thomas et al., 2015). More than 70% of children with ADHD persist with symptoms into adulthood (Sudre et al., 2018). If left untreated, ADHD can result in higher risk of life impairments such as academic failure, family/peer relations, substance abuse, underemployment, and criminality (Biederman et al., 2012).

ADHD patients have deficits in cognitive functions, most prominently in executive functions such as response inhibition, working memory, sustained attention and cognitive switching (Rubia et al., 2007a, Willcutt et al., 2005), as well as in timing (Coghill et al., 2018, Noreika et al., 2013) and reward based functions (Coghill et al., 2018). Functional magnetic resonance imaging (fMRI) studies have consistently shown that ADHD patients have reduced activation in key regions of the fronto-striato-parietal networks that mediate these functions, in particular in the right inferior frontal cortex (rIFC), basal ganglia and medial frontal cortex during cognitive control (Hart et al., 2013, Norman et al., 2016), dorsolateral prefrontal cortex (dlPFC) during working memory (McCarthy et al., 2014), dlPFC, parietal and striatal regions during attention (Hart et al., 2013) and orbitofrontal and ventromedial frontal and striatal regions during reward based functions (Plichta and Scheres, 2014, Rubia, 2018).

Psychostimulants (e.g. methylphenidate, amphetamine) are the most effective treatment, reducing ADHD symptoms in 70% of cases with an effect size of 0.78 up to 12 weeks, and the next most effective approach is non-stimulant medication (e.g. atomoxetine, guanfacine) (Cortese et al., 2018). However, stimulants (and non-stimulants) commonly have side effects on sleep, appetite, growth and cardiovascular systems (Graham et al., 2011) and their long-term efficacy has been questioned (Storebo et al., 2015, Swanson et al., 2018, Zuddas et al., 2018). Meta-analysis of methylphenidate treatment of between 26 and 52 weeks show a more modest clinical effect size of 0.3 (Cortese et al., 2018). In adults with ADHD, treatment length is negatively correlated with efficacy and is associated with higher discontinuation rates, suggesting that medication efficacy does not outweigh side effects (Cunill et al., 2016). These effects may be due to the brain adapting to stimulant medication, as implied through positron emission tomography studies (Fusar-Poli et al., 2012, Wang et al., 2013). This could also explain the low stimulant adherence rates, in particular in adolescence (Cunill et al., 2016). Overall, there is a clear need for alternative treatments for ADHD. Non-pharmacological interventions, including behavioural therapies, exercise (Antshel and Olszewski, 2014, Chronis et al., 2006), diet (Bloch and Mulqueen, 2014, Nigg et al., 2012, Pontifex et al., 2013), and cognitive approaches (Bikic et al., 2015, Dovis et al., 2015), however, have shown only modest efficacy, inferior to that of stimulants (Catala-Lopez et al., 2017, Sonuga-Barke et al., 2013).

Brain-based methods such as neurofeedback (NF), in which patients learn to control their own brain activity via its real-time feedback, may be better suited than pharmacological treatments as they do not have known side effects and can potentially offer longer-term neuroplastic effects (Alegria et al., 2017, Rubia, 2018). Electroencephalography-Neurofeedback (EEG-NF), targeting electrophysiological abnormalities, has been tested in ADHD for over 40 years with relatively moderate efficacy, as shown in recent *meta*-analyses (Cortese et al., 2016, Van Doren et al., 2019).

Real-time fMRI neurofeedback (fMRI-NF), despite its lower temporal resolution (seconds compared to milliseconds), has a superior spatial resolution to EEG-NF (millimetre rather than centimetre) and can target the key brain function deficits that have been established in ADHD over the past 25 years of fMRI research (Rubia, 2018). fMRI-NF enables participants to self-regulate the blood-oxygen level-dependent (BOLD) response of a targeted brain region, or network, through real-time feedback of their brain activity and has shown some promise in psychiatric disorders (Thibault et al., 2016). To date, however, there are only two published fMRI-NF studies in ADHD. In a small underpowered randomised controlled trial, seven adults with ADHD underwent four weekly 1-hour fMRI-NF of dorsal anterior cingulate cortex (dACC), combined with a mental calculation task while six ADHD patients completed the same task in the scanner but were presented with visual cues indicating level of task difficulty instead of fMRI-NF (Zilverstand et al., 2017). Both groups significantly increased dACC activation over the NF runs, including the transfer runs, and improved in an interference inhibition task. Both groups showed trend-level improvements in ADHD symptoms but did not differ from each other. However, only the neurofeedback group showed significantly stronger performance improvement in a sustained attention and working memory task than the ADHD group that received no fMRI-NF, indicative of some positive effects of fMRI-NF of dACC on cognition in adults with ADHD (Zilverstand et al., 2017). A second study, conducted by our lab, tested fMRI-NF in 31 children with ADHD, using 11 fMRI-NF runs of 8.5 min spread across four one-hour fMRI scans. The target group (N = 18) obtained feedback of the rIFC, and the active control group of the left parahippocampal gyrus (lPHG) (Alegria et al., 2017). Both groups showed significantly increased linear activation of their target regions across the fMRI-NF runs, but only the rIFC-NF group showed a transfer effect (learning without the feedback, as a proxy of transfer to real life), and this correlated with decreased ADHD symptoms. Core ADHD symptoms were improved in both groups after fMRI-NF, but with double the effect size in the rIFC-group (assessed approximately one year after the training). Furthermore, only the rIFC-NF group showed trend-level improvements in a sustained attention task after NF training. Relative to the control group, they also showed, following treatment, a significantly greater increase in activation in the rIFC and parietal regions during a motor response inhibition fMRI task. Moreover, the rIFC activation increase after treatment was associated with increased functional connectivity between the rIFC and anterior cingulate cortex (ACC) and caudate, but decreased functional connectivity with regions of the posterior default mode network (DMN), which is associated with mind-wandering (Rubia et al., 2019). This suggests that training to upregulate an isolated brain region, such as the rIFC, was associated with changes in entire fronto-striatal networks of cognitive control, and with anti-correlation with DMN activation. The DMN is typically overactive in ADHD patients and has been suggested to relate to mind-wandering (Bozhilova et al., 2018). A stronger decrease in connectivity between rIFC and areas of the DMN may hence reflect a reduction in abnormally enhanced mind-wandering (Rubia et al., 2019).

To advance the use of fMRI-NF as a potential individualised treatment for ADHD, it will be crucial to understand how many of those with ADHD will respond to the treatment, who the responders are, and what distinguishes them from non-responders. Large heterogeneity of NF response has been observed with EEG-NF in both healthy (Dekker et al., 2014, Enriquez-Geppert et al., 2013, Weber et al., 2011) and clinical populations, including ADHD patients (Doehnert et al., 2008, Drechsler et al., 2007, Kotchoubey et al., 1999, Kouijzer et al., 2013, Liechti et al., 2012, Lubar et al., 1995). A review of 20 EEG-NF studies, which included healthy participants and children with ADHD or Autism Spectrum Disorder (ASD), showed that between 40 and 84% of participants were successful NF-regulators (Alkoby et al., 2018). Similarly, a review of EEG-NF studies in ADHD showed between 65 and 82% success rate in self-regulation (Zuberer et al., 2015). Several studies have attempted to investigate potential predictors of successful EEG-NF learning. A systematic review of EEG-NF and brain-machine interface technology-based (BCI) NF studies in both healthy and clinical populations showed that the ability to concentrate appeared to have a predictive value in NF self-regulation learning, while motivational, mood and personality factors showed relatively moderate importance (Kadosh and Staunton, 2019).

Brain physiology, such as baseline resting state activity, has also been shown to have a predictor value in the success of NF learning and in NF-associated symptom improvement in ADHD. For instance, higher baseline theta activity and higher baseline contingent negative variation was associated with larger improvements in ADHD symptoms after theta/beta EEG-NF training (Gevensleben et al., 2009) or slow cortical potentials (SCP) NF learning, respectively (Wangler et al., 2011). A study using near-infrared spectroscopy showed that pre-training performance and higher left inferior prefrontal cortex (PFC) activation during an executive function Stroop task predicted successful SCP EEG-NF learning in children with ADHD (Okumura et al., 2017). This was interpreted that older children with ADHD may be better suited for NF, as these executive processes and the activation of PFC regions have shown to take longer to mature in children with ADHD compared to their healthy counterparts (Okumura et al., 2017, Sripada et al., 2014).

Given the relative novelty of fMRI-NF and the small number of studies to date, there has been little investigation of predictors of successful fMRI-NF learning. Similarly to EEG-NF, there is large inter-subject variability in fMRI-NF learning ability, including in clinical populations (Chiew et al., 2012, Li et al., 2018, Yoo et al., 2008, Zilverstand et al., 2017, Zweerings et al., 2018). In ADHD, hardly anything is known about predictors of successful brain regulation with fMRI-NF, with only one study having tested for potential predictors of NF learning. The fMRI-NF study of dACC in adults with ADHD found that better accuracy in a 2-back visuospatial working memory task and better inhibitory control in a sustained attention to response task predicted larger improvements in self-regulation learning across sessions in the NF compared to the control group (Zilverstand et al., 2017). Considering the financial costs and complexities required to conduct fMRI-NF studies, it would be extremely beneficial to be able to understand the factors that contribute to better fMRI-NF learning success rates, which will eventually allow future optimal individualised fMRI-NF protocols.

The aim of the current study was therefore to investigate the relationship between the ability to self-regulate brain activity through fMRI-NF and baseline clinical, cognitive, and neurofunctional measures, based on data from our previously published fMRI-NF study in adolescents with ADHD (Alegria et al., 2017). Evidence from EEG-NF studies has demonstrated that brain function measures predict NF learning both in healthy controls (Nan et al., 2018, Reichert et al., 2015, Wan et al., 2014, Weber et al., 2011) and in ADHD patients (Gevensleben et al., 2009, Okumura et al., 2017, Wangler et al., 2011). We therefore hypothesised that stronger baseline activation of fronto-striatal cognitive control regions in the fMRI stop task would be correlated with better fMRI-NF learning. Moreover, we hypothesised that cognitive measures of self-control and attention, which were found to be predictors of self-regulation success with EEG-NF in both healthy and clinical populations (Kadosh and Staunton, 2019) as well as with fMRI-NF in adult ADHD patients (Zilverstand et al., 2017), would predict better fMRI-NF learning. Finally, given the previous NF literature in ADHD (Zilverstand et al., 2017, Zuberer et al., 2018), we expected that clinical behavioural measures would show the weakest association with fMRI-NF learning.

## 2 Materials and methods

The fMRI-NF study design has been previously described in Alegria et al. (2017). Briefly, the randomised controlled trial tested the effects of fMRI-NF of the rIFC in 18 children with ADHD compared to a control group of 13 children with ADHD who underwent fMRI-NF of the lPHG on clinical, cognitive and fMRI measures during a motor response inhibition stop task. All participants completed four 1-hour MRI scans across two weeks and completed an average of 11 fMRI-NF runs of 8.5 min each of their respective training condition. A response inhibition tracking stop task was performed during the first and last fMRI scan sessions, immediately pre- and post-fMRI-NF training administration. Clinical and neurocognitive measures were also recorded, outside the scanner, pre- and post-fMRI-NF training.

### 2.1 Participants

Thirty-one right-handed (Oldfield, 1971) 12–17 year-old boys, with a clinical DSM-5 ADHD diagnosis, combined hyperactive/impulsive and inattentive (N = 27) or inattentive subtypes (N = 4), as assessed by an experienced child psychiatrist and confirmed with the Schedule of Affective Disorders and Schizophrenia for School Age Children-Present and Lifetime version (K-SADS-PL) (Kaufman et al., 1997), were recruited from South London clinics. They met above clinical ADHD threshold on the Conner’s Parent Rating Scale (CPRS-R), a parent rated index of ADHD severity (Conners et al., 1998). The Social Communication Questionnaire (Rutter et al., 2003) was used to screen for ASD. Six boys met above the clinical cut-off score of 15 for potential ASD (2 in the rIFC-group, 4 in the lPHG control group), but a possible ASD diagnosis was ruled out by clinical interview. Children’s Global Assessment Scale was used to assess general function and symptom severity (Shaffer et al., 1983).

Exclusion criteria were IQ < 80 using the Wechsler Abbreviated Scale of Intelligence – Second Edition (WASI-II; Wechsler, 2011), alcohol or substance abuse, neurological or comorbid psychiatric disorders, except for disruptive behaviour disorder, and MRI contraindications. Twenty-four patients received stable psychostimulant administration throughout the fMRI-NF period (methylphenidate: N_rIFC_ = 13, N_lPHG_ = 9, dexamphetamine: N_rIFC_ = 2). Baseline testing started at least seven days after titration. One patient from the control group was medication-naïve, and 3 patients of the rIFC and the control groups each ceased taking medication for at least seven days before baseline testing. The study was approved by the local ethics committee (12/LO/0708) and conducted in accordance with the Declaration of Helsinki. Written informed assent/consent was obtained from each participant/legal guardian. Participants received £20 for each of the fMRI-NF scan visit, and for the post-fMRI-NF neuropsychological assessment, amounting in total to up to £150. Travel expenses were reimbursed. See Table 1 for further demographic details or see Alegria et al. (2017).

**Table 1:** Demographics, medication status, number of fMRI-NF runs completed across participants.

Descriptive Statistics (N = 31)	Mean (SD) or n (%)
*(a) Demographics*	
Age	13.90 (1.58)
WASI-II Full-Scale IQ	103.45 (14.28)
Years in education	9.32 (1.51)
Age of onset of ADHD	6.68 (1.82)
Social Communication Questionnaire	9.24 (5.91)
Oppositional Defiant Disorder	14 (45.16%)
*(b) Medication status*	
Medication naive	1 (3.23%)
On stimulant medication	24 (77.42%)
Off stimulant medication	6 (19.35%)
*(c) fMRI-NF runs*	
Number of runs	11.65 (2.50)
Completed 11 or more runs	21 (67.74%)
Completed all 14 runs	10 (32.26)

*Note.* WASI, Wechsler Abbreviated Score of Intelligence (second edition).

### 2.2 fMRI neurofeedback protocol

The task protocol has been published previously (Alegria et al., 2017). Participants underwent 14 fMRI-NF runs (8.5 min each) in four 1–1.5 h scan visits across 2 weeks. Each fMRI-NF run consisted of alternating blocks of rest (30 s) and activation (50 s). Each run consisted of seven rest blocks and six activation blocks; it started with a rest block during which an image of a dolphin was displayed, while the active NF blocks showed a video-clip of a rocket. Participants were asked to come up with their own strategy to move the rocket towards space and instructions were minimal (e.g. “you can try to concentrate on the rocket” or “try any other method that works for you”). This has been shown to be more effective than explicit instructions (Sulzer et al., 2013) and is commonly used in ADHD EEG-NF studies (Gevensleben et al., 2014, Strehl et al., 2006). Once every repetition time (TR; 2 s) participants received feedback about their brain activity in their target region of interest (ROI) via the rocket-video clip, with the distance travelled in space proportional to their BOLD response (Full details of the feedback signal are given in Alegria et al. (2017)). At the end of each run, a score (0–10), reflecting the distance travelled through space, appeared on the screen (e.g. 7 for 70%), and a monetary incentive (£7 for a score of 7) that corresponded to the best performance in the run was given after the scan. In between runs, the researchers acknowledged participants’ efforts in staying still and reminded them to continue to do so. The participants were also congratulated for the score they obtained after each run. The fMRI-NF performance was acquired for each run, for each participant as another way to measure brain regulation.

Between scan visits, participants were instructed to practice daily brain self-regulation using a cue card with a still-image of the video-clip rocket. After the final fMRI-NF run of the last scan visit, a 5-minute fMRI “transfer” run was conducted. This was identical to the NF run in that the same stimuli were used, but without the actual feedback (i.e. the rocket did not move). The transfer run consisted of four rest and three activation blocks. Transfer runs measure retention of learning and is considered a proximal measure of successful transfer of training strategies to everyday life (see Alegria et al., 2017, Drechsler et al., 2007).

### 2.3 fMRI stop task

An fMRI version of an individually adjusted visual tracking stop task (Alegria et al., 2017, Rubia et al., 2011, Rubia et al., 2005) was completed before the first fMRI-NF run (visit 1) and after the last fMRI-NF run (visit 4). This task measures the ability to unexpectedly suppress a motor response already being triggered by a go-stimulus (Verbruggen et al., 2019). A tracking algorithm changed the time interval between the go-signal and stop-signal onsets according to each participant’s performance on previous trials, resulting in 50% of correctly inhibited trials and 50% of incorrectly inhibited trials. The dependent measure is the stop signal reaction time derived from the stop signal delay at which the subjects managed to inhibit 50% of trials, and the mean reaction time to go trials (stop signal reaction time = mean reaction time to go trials – delay). The contrast of successful stop–go trials assesses inhibitory activation and the contrast of failed stop–go trials assesses error monitoring activation.

### 2.4 fMRI-NF data acquisition and processing

Details of MRI data acquisition, scanning parameters, and the fMRI-NF procedure that were used in the previous study are described in Alegria et al. (2017) and in the supplementary material section 1.1. Briefly, gradient-echo echo planar MR imaging (EPI) and structural data were acquired on a 3 T General Electric MR750 scanner with a 12-channel head coil at the Centre for Neuroimaging Sciences, King’s College London. For the real-time transfer and analysis of the fMRI data, a custom fMRI-NF interface system (Bodurka and Bandettini, 2008) and the Analysis of Functional Neuro Images (AFNI) (Cox, 1996) software were used where fMRI data were pre-processed and corrected for motion in real-time using AFNI. The AFNI anatomical template was used to structurally define the target ROIs (rIFC or lPHG) in Talairach space. The image mask of the pre-selected ROIs was applied to the pre-processed fMRI images and the mean BOLD signal was extracted from each ROI in real-time. For each newly acquired brain volume, AFNI calculated a new set of values for each ROI, and the level of activation was fed back to the participants by means of the moving rocket. The threshold required for the rocket to ascend was continuously updated based on current performance compared to that of the average of the previous rest block (See supplementary material section 1.1 for full details). Participants were informed of the NF delay (~6s), caused by haemodynamic delay and data processing time, before each fMRI-NF run.

### 2.5 fMRI stop task data acquisition

Before the first fMRI-NF run, functional scans for the fMRI tracking stop task were acquired. In each of 38 non-contiguous planes parallel to the anterior-posterior commissure, 200 T2*-weighted MR images depicting BOLD contrasts that covered the whole brain were acquired with TR/TE = 1.800/3ms, with all other parameters matching the fMRI-NF runs that are described in Alegria et al. (2017) and in the supplementary material section 1.1.

### 2.6 Clinical measures

The primary outcome measure was the ADHD rating scale (ADHD-RS), that assesses ADHD symptoms according to DSM-IV and monitors treatment effects (DuPaul et al., 1998). The secondary outcome measure was the CPRS-R ADHD index. Both measures were completed by parents.

### 2.7 Neurocognitive measures

The Maudsley Attention and Response Suppression task battery (MARS) (Rubia et al., 2007a) was used to measure performance on tasks of inhibition, sustained attention, time estimation and temporal discounting. Tasks included a Go/No-Go task (main dependent variable: probability of inhibition), a continuous performance task (CPT; dependent variables: omission and commission errors), a time discrimination task (dependent variable: errors), and an individually adjusted delay discounting task (dependent variable: impulsiveness factor k) (Kekic et al., 2014, Rubia et al., 2009). In addition to the above measures, we also assessed processing speed and intra-subject response variability of reaction time by averaging the mean reaction times and intrasubject response variability, respectively, to go trials in the Go/No-Go task and to target trials in the CPT task.

### 2.8 Data analysis

#### 2.8.1 fMRI-NF data

Data from all 31 participants from the previous study by Alegria et al. (2017) were included in a retrospective fMRI-NF data analysis. The average number of fMRI-NF runs completed for both groups was 11, with only 30% of participants (N_rIFC_ = 4; N_lPHGcontrol_ = 6) completing all 14 runs which were mainly due to technical difficulties and time constraints. Therefore, only the first 11 (or less) fMRI-NF runs were analysed (see Alegria et al., 2017 for details). fMRI-NF brain activation analysis for each participant are identical to the previous study and are described in the supplementary material section 1.2.

The fMRI-NF performance during the fMRI NF training was also recorded and averaged across all runs and a between-group ANOVA was conducted to assess group differences in this performance. The average fMRI-NF performance was significantly higher in the rIFC-NF group (mean = 56.68; SD = 8.64) compared to the lPHG-NF group (mean = 41.63; SD = 9.87) F(1, 29) = 20.373, p < 0.001).

#### 2.8.2 Group differences in linear correlations between brain activation in ROIs and number of fMRI-NF runs

A group comparison of the linear correlation between ROI activation and the number of fMRI-NF runs was conducted as also described in (Alegria et al., 2017), in which a summary statistical map for each run for each group was constructed by averaging the statistical maps of all participants who completed that fMRI-NF run. This resulted in a set of 11 “average maps” per group. The Pearson product-moment correlation coefficient was then computed between the number of fMRI-NF runs and signal change within each group at each voxel in standard space (for more details, see Alegria et al., 2017). Differential effects on linear correlations between the number of fMRI-NF runs and brain activation in the two trained ROIs were then tested. To determine the significance of this difference, the appropriate null distribution was generated by randomly permuting subjects and fMRI-NF run numbers between groups, thus scrambling any group differences. For each permutation, the correlation difference between scrambled groups was calculated and the resulting values were combined over all voxels to produce a whole-brain null distribution of differences in correlation. Testing was then extended to cluster-level and the thresholds were set at p < 0.05 for voxel-level and p < 0.05 for cluster-level, the latter set to yield less than one false positive cluster per map. It was found that the rIFC-NF group had progressively increased activation in two regions of the rIFC (BA44 and BA45) with increasing number of training runs, when compared to the control group, while the lPHG-NF control group showed progressively increased activation in three regions (BA36, BA30 and BA36) with increasing number of sessions when compared to the rIFC-NF group (see Alegria et al., 2017).

#### 2.8.3 Definition of fMRI-NF self-regulation learning across both groups

The most significant cluster of progressively increased activation in each group compared to the other group from the previous study (Alegria et al., 2017) was used for the calculation of fMRI-NF self-regulation learning; rIFC versus lPHG control group (i.e. BA45 in the rIFC-NF group within ROI_rIFC_; peak Talairach co-ordinates (x; y; z;); 43; 33; 16; p < 0.005; 47 voxels), and lPHG versus rIFC group (BA36 in the lPHG-NF group; within ROI_lPHG_; peak Talairach coordinates (x; y; z;); –22; −7; −26; p < 0.01; 27 voxels) (Alegria et al., 2017). For the rIFC-NF group and the control lPHG-NF group, the average BOLD activity of BA45 and BA36, respectively, were extracted from each completed run, Pearson’s correlation coefficients between the number of completed fMRI-NF runs (range 6 to 11 runs) and the average BOLD activity according to the participants’ NF condition (either in BA45 or BA36) were then computed. Greater correlation values corresponded to better linear fMRI-NF self-regulation learning. The fMRI-NF learning values across all participants of their respective target region ranged from −0.96 to 0.84 with a mean of 0.028 and standard deviation of 0.526 (See Figure S1 for scatterplots of individual fMRI-NF learning across NF runs and Figure S2 for the scatterplot of fMRI-NF learning correlation r-values of all participants)

#### 2.8.4 Linear correlations between generic, ROI-independent fMRI-NF self-regulation learning and fMRI activation in the stop task across all participants

The individual subject analysis of stop task brain activation was almost identical to the methods used for the fMRI-NF brain activation analysis described in the supplementary material section 1.2 and in Alegria et al. (2017). Similarly, the standard GLM approach was used to calculate the estimates of the response size to the two stop task conditions against an implicit baseline condition, (successful stop minus go trials; unsuccessful stop minus go trials) at individual subject-level.

To test for a linear correlation between whole-brain activation and ROI-independent generic fMRI-NF learning across all participants, at each voxel in standard space, participants were combined in a single group and the Pearson product-moment correlation coefficient was then computed between the self-regulation learning of each participant (i.e. the correlation between the number of runs and the activation in rIFC or lPHG, depending on the group they were originally assigned to) and the brain activation for the two stop task conditions, the successful stop condition (successful stop–go trials) and the stop failure condition (failed stop–go trials). The correlation coefficients were then recalculated after randomly permuting the fMRI-NF learning values but not the fMRI data. The second step was repeated many times (50 times per voxel, then combining over all voxels) to create a null distribution against which the probability of any particular observed correlation coefficient can be assessed. The analyses were then extended to the 3D cluster level using the procedure described above. In this analysis, less than one error cluster per map was observed at a p-value of p < 0.05 at the voxel level and of p < 0.005 at the cluster level.

#### 2.8.5 Linear correlations between ROI-independent fMRI-NF self-regulation learning and brain activation in the first NF run across all participants

The same analysis detailed in 2.8.4 was conducted to test whether there was a linear correlation between fMRI-NF self-regulation learning and the activation in the first NF run across all subjects.

#### 2.8.6 Linear correlations between generic, ROI-independent fMRI-NF self-regulation learning and clinical and cognitive measures across all participants

Pearson’s linear correlations were computed between the pooled participants’ correlation values in fMRI-NF learning and primary and secondary baseline measures in behavioural (i.e. ADHD-RS, CPRS) and neurocognitive measures (i.e. Go/No-Go task, time discrimination task, temporal discounting task, CPT). The data were assessed for normality with the Shapiro-Wilk test. Spearman’s Rho correlation tests were used instead for non-normal data. Some participants failed to complete all cognitive tasks and questionnaires. Missing data (<5%) were assumed to be completely at random, and missing pre-fMRI-NF training data were replaced by group means (White and Thompson, 2005). Correction for multiple testing was applied using the Benjamini-Hochberg false discovery rate (Benjamini and Hochberg, 1995).

#### 2.8.7 Linear correlations between ROI-specific fMRI-NF self-regulation learning and fMRI activation in the stop task for each group separately

To assess associations between baseline brain activation and ROI-specific learning, we repeated the identical analysis as described in 2.8.4 for each fMRI-NF subgroup separately, i.e. for the rIFC-NF group and the lPHG-NF group at the same voxel p-value of p < 0.05 and p < 0.005 at the cluster level.

#### 2.8.8 Linear correlations between ROI-specific fMRI-NF self-regulation learning and clinical and cognitive measures for each group separately

Pearson’s correlations were computed between clinical and neurocognitive measures and fMRI-NF learning measures for each NF group separately, i.e. between the participants’ fMRI-NF learning correlation values from the active rIFC-NF group and baseline behavioural and neurocognitive measures, and between fMRI-NF learning from the control lPHG-NF group and baseline behavioural and neurocognitive measures.

#### 2.8.9 Categorical analysis of fMRI-NF learners versus non-learners and associations with clinical and cognitive measures

For the categorical analysis, successful fMRI-NF learners were defined as patients who showed a positive correlation (r ≥ 0.15) between the number of NF runs (N = 11) and brain activation in their respective regions that most progressively increased during the NF in their group relative to the other group (BA 45 for active; BA 36 for controls).

Between group ANOVAs were then conducted to compare fMRI-NF learners and non-learners in clinical and neurocognitive outcome measures. In order to assess whether baseline clinical or cognitive measures predicted fMRI-NF learner status, we additionally conducted a logistic regression analysis between learners and non-learners. Independent t-tests were first conducted between learners and non-learners in primary baseline measures in behavioural (i.e. ADHD-RS, CPRS) and neurocognitive measures (i.e. Go/No-Go task, time discrimination task, temporal discounting task, CPT). Baseline measures that differed significantly between groups were then entered into the binary logistic regression model. This was conducted so that not all variables were added to the model in the first instance since adding too many variables would lead to reduced statistical power in addition to increasing the risk of detecting false positives (Sperandei, 2014).

## 3 Results

### 3.1 Correlation between ROI-independent fMRI-NF learning and brain activation during the stop task across all participants

**Fig. 1:**
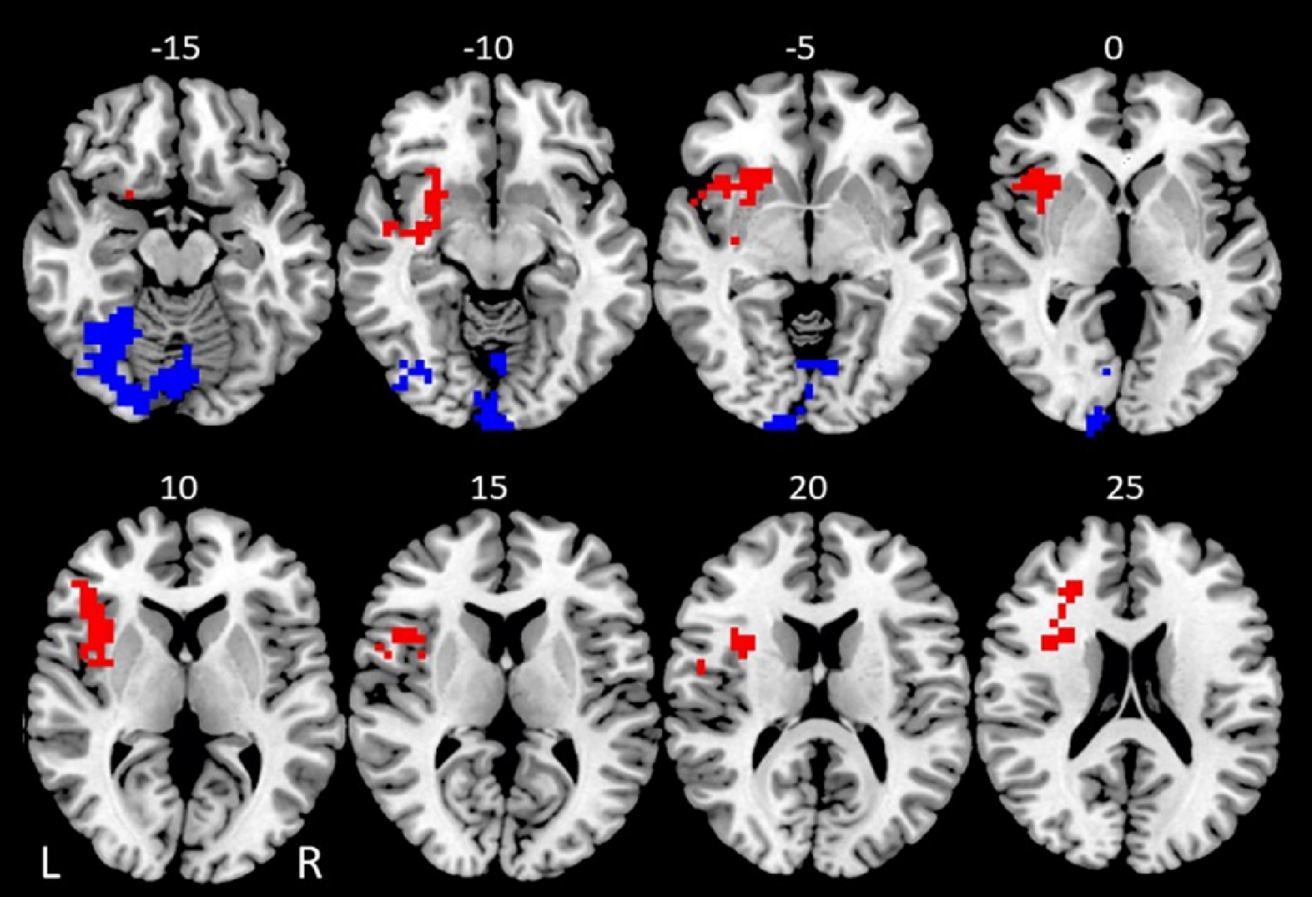
Axial slices showing linear correlations across all subjects between fMRI-NF regulation learning scores and brain activation during successful stop–go trials during the baseline stop task at false positive error-corrected voxel-level of p < 0.05, and cluster-level of p < 0.005 (yielding < 1 false positive cluster per map). The brain cluster in red corresponds to the significant positive correlation between brain activation and fMRI-NF learning scores, and the brain cluster in blue corresponds to the significant negative correlation between activation and fMRI-NF learning scores. The right side of the image corresponds to the right side of the brain. Axial slices are shown in mm distance from the anterior-posterior-commissure. (For interpretation of the references to colour in this figure legend, the reader is referred to the web version of this article.)

The whole-brain correlation analysis between fMRI-NF regulation learning and brain activation during the successful stop–go trials of the stop task (at a voxel-level p < 0.05 and cluster level p < 0.005) revealed progressively enhanced activation with increasing fMRI-NF learning values in a cluster comprising the left inferior and middle frontal cortices, left anterior insula, putamen and nucleus accumbens (see Table 2A & Fig. 1 (cluster in red)). Significant negative correlation between fMRI-NF self-regulation learning and brain activation was observed in the left cerebellum and in left inferior temporal-occipital regions (see Table 2B & Fig. 1A (cluster in blue)). No significant correlation was observed during the failed stop trials.

**Table 2:** Significant positive and negative correlation between brain activation during successful stop–go trials in baseline stop task across participants with fMRI-NF performance.

Brain Regions	Brodmann's Area (BA)	Peak Talairach Co-ordinates (x;y;z)	Cluster Size (voxels)	Cluster p-value^a^
A. Successful Stop - Go Trials: positive correlation				
L inferior/middle frontal cortex/anterior insula/ putamen/nucleus accumbens	BA45/47/46	–32; 22; 3	203	0.001801
B. Successful Stop - Go Trials: negative correlation				
L cerebellum/inferior temporal/fusiform/occipital gyri	BA20/37/17/18/19	−25; −70; −20	185	0.002178

^a^Statistical thresholds were set at p < 0.05 for voxel-level and p < 0.005 for cluster level, resulting in less than one false positive cluster per map.

### 3.2 Linear correlations between fMRI-NF self-regulation learning scores and brain activation in the first NF run

No significant correlations were found between fMRI-NF learning scores across all participants and brain activation during the first fMRI-NF run.

### 3.3 Clinical and neurocognitive measures associated with generic, ROI-independent fMRI-NF learning across all participants

No significant correlations were observed between any baseline primary clinical measures and the fMRI-NF learning values across all the participants (see Table 3A). For the baseline neurocognitive measures, there was a negative correlation between fMRI-NF learning and combined reaction times to target trials in the CPT and go trials in the Go/No-Go tasks (r = -0.447, p = 0.012). A positive correlation was also shown between fMRI-NF learning and k median (r = 0.377, p = 0.037) in the delay discounting task. There was a trend-level negative correlation between the probability of inhibition in the Go/No-Go task and fMRI-NF learning (r = -0.352, p = 0.052). However, none of the findings survived correction for multiple testing using the Benjamini & Hochberg false discovery rate (see Table 3).

**Table 3:** Correlation between baseline primary clinical and neurocognitive measures with fMRI-NF learning scores across all participants.

Baseline clinical measures	Mean (SD)	Correlation r value (df = 29)	P-value (2-tailed)	Adjusted P-value^a^
*ADHD-Rating Scale*				
Total score	37.16 (10.13)	0.062	0.74	
inattention	20.29 (4.47)	0.113	0.546	
hyperactivity/impulsivity	16.87 (6.39)	0.019	0.917	
*Conner’s Parent Rating Scale*				
ADHD Index score	14.81 (4.29)	0.14	0.452	
DSM-V inattention	81.16 (8.53)	−0.113	0.545	
DSM-V hyperactivity/impulsivity	85.48 (9.13)	0.215	0.245	
**Baseline neurocognitive measures**				
*Go/No-Go Task*				
Probability of inhibition (%)	62.48 (19.02)	−0.352	0.052	0.242
*Continuous Performance Task (CPT)*				
Omission errors (%)	8.29 (6.94)	0.217	0.241	
Commission errors (%)	1.13 (1.43)	0.149	0.423	
*Delay Discounting*				
k median	0.015 (0.014)	0.377*	**0.037**	0.242
*Time Discrimination Task*				
Total correct	77.13 (16.63)	−0.05	0.788	
*Stop Task*				
Stop signal reaction time (ms)	116.71 (168.74)	−0.009	0.962	
*RT combined (CPT, Go-No-Go)*	369.11 (40.83)	−0.447*	**0.012**	0.168
*Intra-subject co-efficient of variance combined (CPT, Go-No-Go)*	0.25 (0.06)	0.202	0.275	

*Note.* RT combined, combined mean reaction time to targets during Go/No-Go task and Continuous Performance task.

*Significance level < 0.05 for unadjusted p-values.

**^a^**Benjamini-Hochberg False Discovery Rate adjusted p-value to correct for multiple testing.

#### 3.3.1 Correlation between the number of completed NF runs and clinical and neurocognitive measures

Mostly due to technical errors (e.g. with the MRI scanner, real-time fMRI-NF software etc.) and time constraints, not all participants completed all NF runs (Alegria et al., 2017). Nevertheless, we conducted Pearson’s correlation analyses to test whether baseline primary and secondary clinical and cognitive measures were associated with the number of completed NF runs. No significant correlations were found between the number of runs and any measures (strongest r(29) = 0.282; smallest p = 0.125; p = n.s.; see Table S1 in supplementary materials).

### 3.4 Correlation between ROI-specific fMRI-NF learning and brain activation during the stop task separately for each of the two fMRI-NF groups

**Fig. 2:**
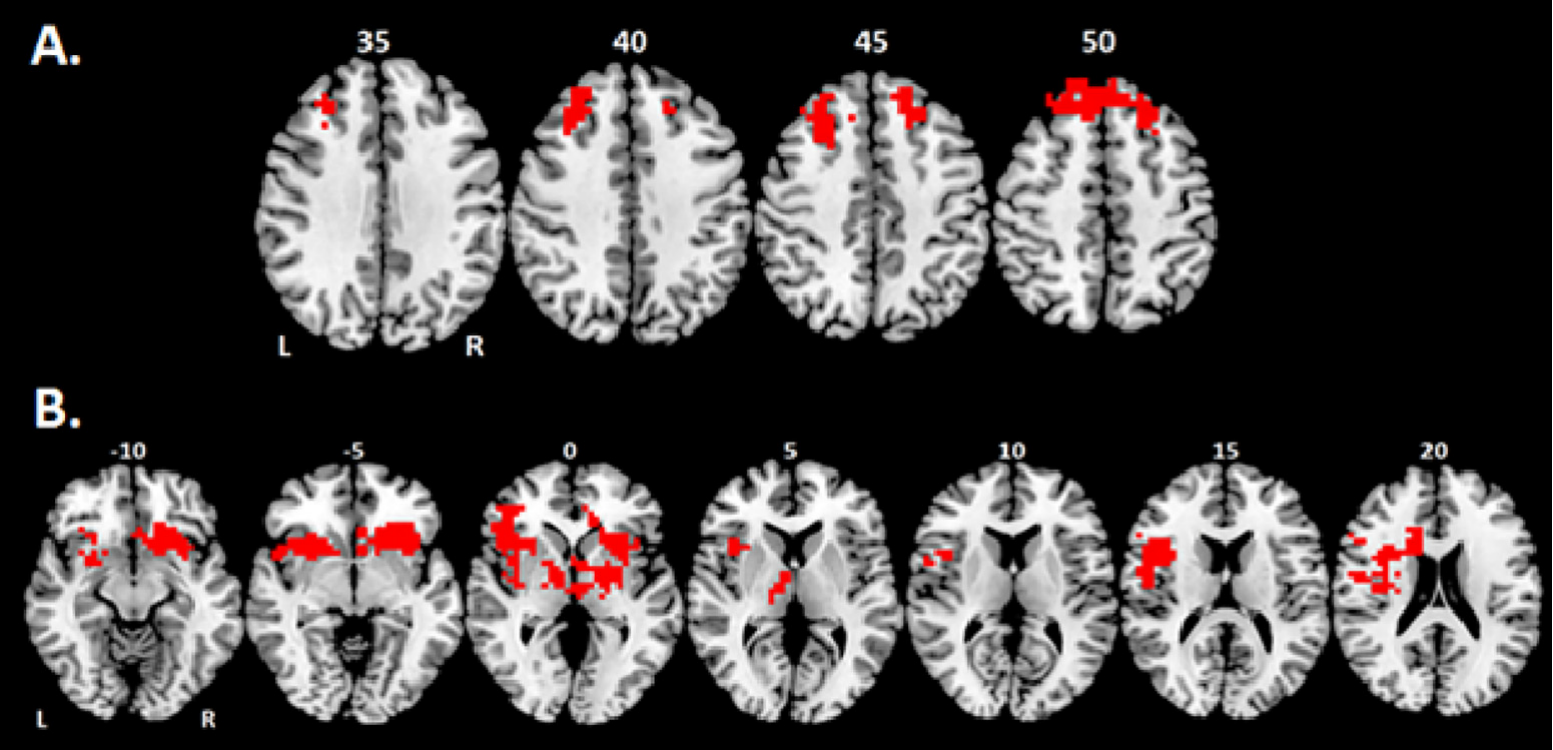
Axial slices showing whole brain linear correlation between brain activation in the baseline stop task (successful stop - go trials) and self-regulation learning scores of **A)** rIFC activation in the rIFC-NF group and of **B)** lPHG activation in the lPHG-NF group; both at false positive error-corrected voxel-level p < 0.05, and cluster-level p < 0.005 (yielding < 1 false positive cluster per map). The brain clusters in red correspond to the significant positive correlation between stop-task related brain activation across the whole brain and fMRI-NF learning. There were no significant negative correlations between stop task-related brain activation and fMRI-NF learning in either of the two groups. The right side of the image corresponds to the right side of the brain. Axial slices are shown in mm distance from the anterior-posterior-commissure. (For interpretation of the references to colour in this figure legend, the reader is referred to the web version of this article.)

The whole-brain correlation analysis between rIFC-NF regulation learning in the active group and their brain activation during the successful stop–go trials of the fMRI stop task (at a voxel-level p < 0.05 and cluster level p < 0.005) revealed progressively enhanced activation with increasing fMRI-NF learning values in a cluster comprising the left and right superior and middle frontal cortices (Table 4A & Fig. 2A). Significant positive correlation between lPHG-NF learning and stop task-related brain activation was observed in a cluster of the left orbitofrontal/inferior frontal areas, insula, anterior cingulate, pre-motor cortex, and reaching subcortically into the putamen, caudate, globus pallidum and thalamus. The other cluster comprised similar regions of the right hemisphere including orbitofrontal/inferior frontal areas, insula, anterior cingulate, putamen, caudate, globus pallidum, thalamus, and areas of the superior temporal lobe (Table 4B & Fig. 2B). There were no significant negative correlations between stop-task related brain activation and fMRI-NF learning in either of the two groups.

**Table 4:** Significant positive correlation between brain activation across the whole brain during successful stop–go trials in the baseline stop task and fMRI-NF learning scores in rIFC in the rIFC-NF group and fMRI-NF learning scores in lPHG in the lPHG-NF group.

Brain Regions	Brodmann's Area (BA)	Peak Talairach Co-ordinates (x;y;z)	Cluster Size (voxels)	Cluster p-value^a^
A. rIFC-NF Group				
L&R superior/middle frontal cortex	BA8/9	−7; 37; 46	152	0.001515
B. lPHG-NF Group				
L orbitofrontal/inferior frontal cortices, insula, anterior cingulate, putamen, caudate, globus pallidum, thalamus, premotor cortex	BA47/44/45/6/	−36; 19; −10	313	0.000359
R insula, orbitofrontal/inferior frontal cortices, anterior cingulate, superior temporal, putamen, caudate, globus pallidum, thalamus,	BA47/44/32/25/22	22; 26; −10	243	0.000737

^a^Statistical thresholds were set at p < 0.05 for voxel-level and p < 0.005 for cluster level, resulting in less than one false positive cluster per map.

### 3.5 Clinical and neurocognitive measures associated with ROI-specific fMRI-NF learning for each group separately

No significant correlations were found between fMRI-NF learning in the active rIFC-NF group or the control lPHG-NF group in any baseline behavioural or neurocognitive measures with the exception of (faster) mean reaction time to targets during Go/No-Go and CPT (RT combined) being significantly negatively correlated with (better linear) fMRI-NF learning in the control lPHG-NF group. This survived correction for multiple testing (FDR) (r (11) = -0.757; p-adjusted = 0.042).

### 3.6 Categorical analysis of fMRI-NF learners versus non-learners and clinical and cognitive measures

For the categorical analysis, successful fMRI-NF learners were defined as patients who showed a positive correlation (r ≥ 0.15) between the number of NF runs (N = up to 11) and brain activation in their respective regions that most progressively increased during the NF in their group relative to the other group (BA45 for active; BA36 for controls). The correlation analysis revealed 15 successful learners and 16 non-learners out of 31 participants, resulting in a percentage of self-regulation learning of 48.4% (Figs. S1 & S2, supplementary materials).

We then tested whether learners and non-learners differed in pre- and post-fMRI-NF training changes in clinical and neurocognitive measures. Within-group comparisons showed that ADHD symptoms in both the fMRI-NF learner group and the non-learner group decreased significantly from pre- to post-fMRI-NF, for all primary and secondary outcomes measures, except for only a trend-wise reduction in ADHD-RS hyperactivity/impulsivity subscale in the non-learners (Table S2 in supplementary materials).

Comparisons between successful fMRI-NF learners and non-learners revealed a significant effect of time showing decreased symptoms in primary (ADHD-RS total score, F(1,29) = 15.76, p < 0.001, d = 0.63; ADHD-RS Inattention subscale, F(1,29) = 15.67, p < 0.001, d = 0.70; ADHD-RS Hyperactivity/Impulsivity subscale, F(1,29) = 10.37, p = 0.003, d = 0.47) and secondary outcome measures (CPRS-R ADHD Index, F(1,29) = 17.00, p < 0.001, d = 0.72; CPRS-R DSM-5 inattention, F(1,29) = 14.67, p = 0.001, d = 0.955; CPRS-R DSM-5 hyperactivity/impulsivity, F(1,29) = 9.94, p = 0.004, d = 0.352) in both groups, but there were no group or group by time interaction effects (F(1,29) < 2.08, p > 0.16).

For the neurocognitive measures, fMRI-NF learners showed reduced CPT commission errors (percentage) post- versus pre-fMRI-NF with a medium effect size (F(1,29) = 4.83, p < 0.045, d = 0.49) only. No other neurocognitive performance effects were observed within groups (Table S3 in supplementary materials). Between group comparisons showed no significant effect of time in any measures (F (1, 29) < 3.73), p > 0.06). There was a significant effect of group in the combined mean reaction time to targets in the Go/No-Go and CPT (F (1,29) = 7.73, p = 0.009, d = 0.12), with learners being faster than non-learners. No group by time interaction effects were significant (F (1,29) = 2.23, p > 0.15).

In the cognitive baseline measures, only mean reaction time to targets in the Go/No-Go and CPT combined (RT combined; t(df = 29) = 2.839, p = 0.008), and k-median (t(df = 29) = -2.28), p = 0.03) in the delay discounting task differed significantly between groups. Mean reaction time in the Go/No-Go and CPT combined was faster in learners, and non-learners had steeper discounting than non-learners. The above mentioned measures that differed significantly between groups (RT combined and k-median in delay discounting) were inputted into the logistic regression analysis. The analysis revealed that only RT combined was a significant predictor of successful NF regulation learning in which faster RT was associated with better fMRI-NF learning (Odds ratio: 0.967, 95% CI: 0.937 – 0.997, p = 0.033).

## 4 Discussion

The aim of this study was to explore the relationship between fMRI-NF self-regulation learning in adolescents with ADHD and baseline scores in neurofunctional, clinical, and neurocognitive measures in order to establish fMRI-NF learning predictors. Similar to previous EEG-NF and fMRI-NF studies in both healthy and clinical population, there was relatively large heterogeneity in the NF regulation capability across participants, with self-regulation learning values ranging from −0.96 to 0.84; where higher positive correlation values indicating better linear fMRI-NF learning (Alkoby et al., 2018, Okumura et al., 2017, Zuberer et al., 2015). The categorical analysis of learners and non-learners showed that the percentage of successful fMRI-NF learners was 48.4% (N = 15 out of 31), similar to what has been found in EEG-NF studies in healthy participants and in ADHD patients (Alkoby et al., 2018, Okumura et al., 2017, Zuberer et al., 2015). The correlation analyses revealed that, in the baseline fMRI stop task, better fMRI-NF learning was associated with enhanced activation during successful stop trials in a large cluster comprising the left inferior and middle frontal cortices, anterior insula, putamen and nucleus accumbens, while poorer fMRI-NF learning was associated with enhanced temporo-occipital-cerebellar activation. However, no significant correlation was observed with activation during the failed stop task, suggesting that only networks during successful inhibitory self-control and not error monitoring activation patterns are associated with fMRI-NF learning. At the cognitive level, only in the categorical analysis, processing speed during sustained attention and inhibition was predictive of successful NF learning while it did not survive correction for multiple testing in the correlation analysis between cognitive measures and fMRI-NF learning. No associations were found between fMRI-NF learning and clinical measures in either the correlational or categorical analyses.

Taken together, these findings indicate that functional neuroimaging data are stronger predictors of fMRI-NF learning than cognitive or clinical measures. They show that better self-regulation learning during fMRI-NF in adolescents with ADHD is associated with increased recruitment of left fronto-insular-striatal cognitive control regions during an inhibitory self-regulation task. In contrast, poorer fMRI-NF regulation learning was associated with increased posterior temporo-occipital-cerebellar activation during the fMRI stop task. This activation pattern of increased left fronto-striatal and reduced posterior temporo-parietal regional recruitment during a task of self-control in better fMRI-NF self-regulators may be reflective of a more mature activation pattern. Fronto-striatal cognitive control networks and posterior temporo-occipital regions have been shown to be increasingly and decreasingly recruited, respectively, with increasing age between childhood and adulthood (Rubia, 2013), in particular during motor response inhibition tasks (Christakou et al., 2009, Rubia et al., 2013, Rubia et al., 2007b, Rubia et al., 2006). Moreover, inferior fronto-striatal regions are typically under-activated in children with ADHD relative to age-matched healthy children (Hart et al., 2013, Lukito et al., 2020, Norman et al., 2016, Rubia et al., 2005). In addition, there is evidence for a functional maturation delay in ADHD patients in the development of cognitive control and attention networks (Sripada et al., 2014). Thus, our findings could potentially suggest that children with ADHD with more mature activation patterns in cognitive control regions may be better at self-regulation learning than children who show a more immature activation pattern in posterior brain regions.

The association of enhanced activation of the left inferior fronto-striatal cognitive control regions during successful inhibition associated with better fMRI-NF learning suggest that fronto-striatal cognitive control regions play an important role in mediating fMRI-NF learning. This finding in adolescents with ADHD is similar to the results from an fMRI-NF study in healthy adults, in which better NF-induced regulation of primary motor cortices during a kinesthetics motor imagery task was associated with enhanced activation of bilateral middle frontal cortex, insula, basal ganglia, thalamus, and premotor cortex during fMRI-NF training (Chiew et al., 2012). Our findings also extend findings from an EEG-NF study in children with ADHD in which stronger baseline activation of left inferior PFC measured with near-infrared spectroscopy during a matching Stroop task predicted SCP EEG-NF regulation success, suggesting that NF training success may depend on the maturity of left PFC activation mediating executive processes in ADHD patients (Okumura et al., 2017).

Our findings that increased fronto-striatal cognitive control activation is related to better fMRI-NF learning in ADHD patients also extend recent *meta*-analytic findings of 12 fMRI-NF studies in healthy controls that included 9 different NF target ROIs. The *meta*-analysis revealed a common network which was consistently increased during fMRI-NF training, independent of the region that was trained to be regulated. This network comprised the inferior and dlPFC, ACC, anterior insula, basal ganglia, temporo-parietal and visual association regions; and were thus proposed as regions that mediate generic brain self-regulation processes (Emmert et al., 2016). These regions form part of the cognitive control network (Sripada et al., 2014), and overlap with the areas which we found to be increased in activity with better fMRI-NF learning in adolescents with ADHD during the fMRI stop task. The regions highlighted by our study also overlap with the proposed fMRI-NF regulation networks described in a review by Sitaram et al. (2017); the authors proposed three networks: the control network comprising the dlPFC, posterior parietal cortex and, thalamus; the reward processing network comprising the ACC, anterior insular cortex and ventral striatum; and the learning network which comprises the dorsal striatum.

Although the right inferior frontal cortex together with the anterior insula and striatal regions have more typically been associated with inhibitory motor control (Aron et al., 2014, Guo et al., 2018, Rubia et al., 2003), the left inferior frontal cortex and striatal regions have also been implicated in motor inhibition in fMRI and lesion studies, in particular in children (Criaud and Boulinguez, 2013, Hampshire et al., 2010, Rae et al., 2014, Rubia et al., 2013, Rubia et al., 2007b, Sebastian et al., 2016, Swick et al., 2008). Some functional connectivity studies have even argued for a stronger role for the left than the right IFC in mediating inhibition, together with the pre-supplementary motor area (SMA) (Duann et al., 2009, Zhang et al., 2012). The left IFC has also been argued to mediate attentional target detection processing and to kick-start the inhibitory process via its attention processing role, which then initiates the inhibitory process via its connection to the pre-SMA (Chao et al., 2009). The left IFC, anterior insula and striatum have also been implicated in wider cognitive control functions, including interference inhibition and switching, and not only motor response inhibition (Christakou et al., 2009, Cole et al., 2014, Hugdahl et al., 2015, Niendam et al., 2012). It is hence plausible that regions that mediate generic cognitive control, rather than those that mediate motor inhibitory control specifically, are implicated in brain self-regulation.

The bilateral IFC are also part of the ventral attention system (Corbetta et al., 2008, Shulman et al., 2009). Several fMRI studies using stop task manipulations have shown that the bilateral ventral IFC attention system together with the pre-SMA and inferior parietal lobes are activated during attention processing or attentional preparatory processes to the behaviourally relevant rare stop trials (Chao et al., 2009, Duann et al., 2009, Hampshire et al., 2010, Hu and Li, 2012, Zhang and Li, 2012). It is hence also possible that top-down attentional processes are associated with better brain self-regulation capacity.

Interestingly, the left inferior and middle frontal areas, together with the putamen and nucleus accumbens, have also been implicated in cognitive control learning and planning processes (Brovelli et al., 2011, Grahn et al., 2008, Kaller et al., 2010, Liljeholm and O'Doherty, 2012). A review of both comparative and human NF-related studies proposed that the basal ganglia play a key role in the success of NF training (Birbaumer et al., 2013) as there is strong evidence of cortico-basal-ganglia loop involvement in self-regulation and skill learning (e.g. Halder et al., 2011, Hinterberger et al., 2005, Koralek et al., 2012). Our findings thus imply that the more skilled ADHD fMRI-NF learners may have a better ability to engage their cognitive control network during a cognitive control task to begin with. This is associated with superior fMRI-NF learning skills, presumably because the same regions that mediate inhibitory control are also involved in fMRI-NF self-regulation.

Interestingly, the test for associations between fMRI-NF learning and stop task activation in each of the two fMRI-NF groups separately, showed that ventrolateral PFC, insula and striato-thalamic activation was associated with fMRI-NF learning in the lPHG group while a more dorsal prefrontal activation cluster was associated with NF learning in the rIFC-NF group. As mentioned above, both IFC and dlPFC are associated with fMRI-NF learning in the *meta*-analysis of Emmert et al (2016) and both regions were associated with generic fMRI-NF learning in our analysis across all participants. It seems that successful learning of rIFC-NF activation benefitted more from dlPFC baseline activation, while successful learning of lPHG activation benefitted more from ventrolateral PFC baseline activation. The left dlPFC is a key region of learning (Brovelli et al., 2011, Grahn et al., 2008, Kaller et al., 2010, Liljeholm and O'Doherty, 2012) and exerts top-down control over other prefrontal regions such as IFC and orbitofrontal cortex within a caudo-rostral prefrontal hierarchy (Dosenbach et al., 2008, Milham et al., 2003, Silton et al., 2010). A strong baseline dlPFC activation may hence be crucial for self-regulation of inferior frontal regions. The orbitofrontal cortex and insular regions are closely connected to the parahippocampal gyrus and thus, may be important for self-regulation of this region (Suzuki, 2009). It has furthermore been shown that posterior and smaller regions are more difficult to self-regulate compared to more anterior, higher-level association areas. One study showed that within 4 runs in an fMRI-NF session, the anterior insula could be successfully upregulated but not middle parahippocampal regions; however, this study used affective probes and strategies targeting anterior insula and not parahippocampal activation (Lawrence et al., 2014). Other studies demonstrated that posterior regions such as lower visual areas compared to the higher visual and inferior parietal areas are more difficult to self-regulate (Harmelech et al., 2015), and a small pilot study found that the posterior as opposed to the rostral anterior cingulate cortex (Guan et al., 2015), could not be successfully self-regulated. In our own study, only the rIFG-NF group had a transfer effect and showed a significant difference between the last and first run in rIFC activation, while the control group did not show such effects and only showed a linear activation increase in lPHG (which was relatively weaker than the activation increase in rIFC) (Alegria et al., 2017). This was also reflected in the scores in the fMRI-NF performance during the training which were significantly higher in the rIFC compared to the lPHG group. Therefore, if the lPHG-NF training is more challenging than the rIFC-NF training, demanding superior self-regulation skills, then this could potentially explain the larger bilateral ventrolateral PFC-striato-thalamic activation clusters associated with better fMRI-NF learning in the lPHG group. In conclusion, it is hence possible that stronger baseline ventral inferior fronto-striatal cognitive control activation is needed to self-regulate a smaller region that is more difficult to self-regulate than frontal regions, while baseline dlPFC activation is more instrumental for frontal self-regulation capacity.

Conversely, enhanced activation of the inferior temporal and occipital regions, and of the cerebellum, during the stop task, in association with poorer fMRI-NF learning, could reflect a more immature activation pattern. During development from childhood to adulthood, regions of inhibitory and cognitive control, in particular the inferior PFC, dlPFC and the basal ganglia, are progressively more recruited with increasing age, while earlier-developing visual-spatial (posterior occipital) and cerebellar regions are recruited more in younger subjects (Christakou et al., 2009, Rubia, 2013, Rubia et al., 2013, Rubia et al., 2007a, Rubia et al., 2006). An inverse interaction between age and ADHD has been shown in large resting state fMRI data in cognitive control and attention networks, suggesting that ADHD patients have a maturational delay of brain function, with the typically age-related progressive development of cognitive control and attention networks being immature (Sripada et al., 2014). It is hence possible that the poorer fMRI-NF learners were more immature in their brain function development, and thus showed less fronto-striatal cognitive control activation together with abnormally increased posterior temporo-occipital and cerebellum activation, which prevented them from learning to self-control their brain activation via fMRI-NF more easily.

An alternative explanation for the enhanced activation of inferior temporal and cerebellar regions in association with poorer self-regulation learning during the fMRI stop task, could be that it reflects increased DMN activation, which these regions have been associated with (Buckner et al., 2011, Krienen and Buckner, 2009, Kucyi et al., 2015). The DMN refers to a “task-negative” network comprising the posterior cingulate cortex (PCC), precuneus, ventromedial frontal regions and inferior temporal and parietal areas. These regions are mostly activated during rest, are thought to reflect internally oriented and task-irrelevant thought processes (e.g. mind wandering), and are supressed during goal-oriented tasks (i.e. they anti-correlate with “task positive networks”) (Raichle et al., 2001). Behavioural studies have shown that ADHD patients have significantly more mind-wandering than healthy controls (Bozhilova et al., 2018, Mowlem et al., 2016, Van den Driessche et al., 2017). fMRI studies have shown that ADHD patients compared to healthy controls have less deactivation of anterior and/or posterior DMN regions during cognitive tasks, especially during attention or executive function tasks with progressively increasing task difficulties, which is associated with poorer attention task performance and enhanced distractibility (Christakou et al., 2013, Metin et al., 2014, Rubia, 2018).

Interestingly, in our current study, only the contrast of successful inhibition was associated with NF self-regulation learning and not the contrast of performance monitoring. This suggests that inhibitory-related brain activation is more relevant to self-regulation skills than error monitoring networks.

At the cognitive performance level, the association between better fMRI-NF learning capacity and faster mean reaction times during a sustained attention and an inhibition tasks across all participant did not survive correction for multiple testing. However, the categorical analysis did show that learners were significantly faster than non-learners overall and the predictor analysis showed that processing speed during inhibition and sustained attention predicted fMRI-NF learning. Furthermore, when investigating the relationship between baseline measures and fMRI-NF learning in the two NF groups separately, the control group showed significant correlation between better learning and faster mean reaction time to targets. The association between NF learning and processing speed during attention and inhibition tasks is in line with prior findings from EEG and fMRI-NF studies in both healthy and clinical populations, including ADHD patients (Daum et al., 1993, Hammer et al., 2012, Zilverstand et al., 2017), that better baseline sustained attention abilities are important for NF learning. ADHD patients have consistently been shown to have slower mean reaction time in cognitive task performance in comparison to healthy controls (Coghill et al., 2018, Kofler et al., 2013, Levy et al., 2018, Losier et al., 1996) presumably reflecting slower processing speed and information processing. In fact, more recent findings indicate that slower reaction time and increased reaction time variability could also be a reflection of attentional lapses and poorer vigilance (Gmehlin et al., 2016, Hervey et al., 2006, Leth-Steensen et al., 2000), potentially reflecting enhanced mind-wandering (Castellanos et al., 2005, Epstein et al., 2011, Lee et al., 2015, Leth-Steensen et al., 2000).

It is interesting to note that the left dlPFC is a key mediating region of processing speed (Jacobs et al., 2013, Motes et al., 2011, Motes et al., 2018), given that both left PFC activation and processing speed were found to be predictors of better fMRI-NF learning in our study. Thus, our finding of faster mean reaction times in both the CPT and the Go/No-Go task associated with better fMRI-NF learning may imply that better self-regulation ability may be related to faster processing speed and information processing skills. Such an association seems plausible since fMRI-NF is an operant conditioning learning procedure, and it has been shown that information processing speed can play an important role in higher executive functioning such as reasoning and learning (Chiaravalloti et al., 2003, Kail et al., 2016, Takeuchi and Kawashima, 2012). Although the correlation analysis showed associations between fMRI activation during the stop task and self-regulation learning, we found no association between the stop task performance and regulation learning skills. We have shown previously in ADHD that fMRI is more sensitive than performance data to detect differences relative to healthy controls (Rubia et al., 1999, Rubia et al., 2005, Smith et al., 2006).

We also found no association between the activation during the first NF run and fMRI-NF learning. The first NF run, however, is likely to be confounded by scanner anxiety and insecurities with respect to the novel aspects of fMRI-NF self-regulation learning and may hence not be representative. Moreover, there was no association between clinical measures and NF self-regulation learning. This echoes previous findings from EEG-NF (Kadosh and Staunton, 2019, Zuberer et al., 2018) and fMRI-NF (Zilverstand et al., 2017) studies that also found little or no association. This suggests that clinical measures may not be as sensitive as neuroimaging and neurocognitive measures in detecting the contributing factors influencing fMRI-NF regulation learning. Together with our findings, this therefore suggests that, in future studies, it may be more informative to examine neuroimaging and neurocognitive measures rather than clinical measures as potential predictors of success in fMRI-NF learning.

Lastly, the number of completed NF runs was not associated with baseline clinical or cognitive measures, suggesting that ADHD clinical or cognitive severity was not a reason or confounder of the completion of NF runs. This was more likely caused by the technical issues that occurred including NF software problems and scanner hardware problems.

## 5 Limitations

As already mentioned above, a limitation is the small sample size which did not allow us to test for learners and non-learners in each group separately. Another limitation is the definition used to measure fMRI-NF regulation learning and the categorical classification of tfMRI-NF learners and non-learners (i.e. defining learners as those with a positive correlation of r > 0.15 between activity of their respective significantly increased target region with the number of completed NF runs) which could be considered arbitrary. However, previous EEG-NF studies have employed similar definitions to ours (e.g. Kouijzer et al., 2013, Lubar et al., 1995). Moreover, there is a large heterogeneity in the type of definitions used across NF studies. NF reviews have highlighted the heterogeneity of the type of definitions used for successful regulation such as “cross-session learning” which refer to brain changes across all (Enriquez-Geppert et al., 2014, Janssen et al., 2017, Lubar et al., 1995) or at certain time-point NF sessions e.g. 1st, 5th, 6th, 13th NF sessions; Gevensleben et al. (2014)) or the transfer session (Doehnert et al., 2008). Alternatively, successful regulation can also be defined by “within-session learning” which considers brain activity changes within a single NF session (Zuberer et al., 2015). We could not examine “within-session learning” as some of our participants did not undergo more than two NF runs in some sessions due to various issues (e.g. compliance or technical issues with the scanner and/or real-time NF software). Still, there is currently no clear consensus on the best definition of successful brain self-regulation (Alkoby et al., 2018), which may be due to the fact that there is also a variation in NF study designs, such as number of NF runs, sessions and length of the blocks. Thus, this makes it difficult to compare and confirm our findings with previous NF studies investigating regulation success. Future NF studies are needed to confirm the optimal way to quantify successful self-regulation for more sound comparisons and discussions across NF studies.

## 6 Conclusions

In summary, the current study shows that better fMRI-NF self-regulation learning in adolescents with ADHD was associated with increased activation during inhibitory control in a left inferior fronto-insular-striatal cognitive control network and with decreased activation in posterior temporo-occipital-cerebellar regions, presumably reflecting a more mature activation pattern of cognitive control. Our study thus suggests that adolescents with ADHD with a more mature pattern of fronto-striatal cognitive control activation to start with may be better suited for fMRI-NF. Our findings also show that neurofunctional measures appear to provide better predictor value of fMRI-NF learning in ADHD patients than either clinical or cognitive behavioural measures. This can help guide future research, and our clinically relevant results can lead to the eventual optimisation of fMRI-NF protocols in ADHD patients to increase the success rates of NF training. This would also make it possible to predict which patients will respond and which will not respond to fMRI-NF and hence help with precision medicine.

## 7 Credit author statement

Study conceptualisation: KR, VG, SLL,; Data acquisition: AA; Software: GJB, VG; Data analysis: SLL, KR; interpretation of data: MC, SLL, KR; Drafting of the manuscript: SLL, KR; Critical revision of the manuscript for important intellectual content: all authors.

## CRediT authorship contribution statement

**Sheut-Ling Lam:** Conceptualization, Data curation, Formal analysis, Investigation, Methodology, Visualization, Writing - Original draft, Writing - Review & editing. **Marion Criaud:** Supervision, Writing - Review & editing. **Analucia Alegria:** Data curation, Investigation. **Gareth J. Barker:** Software, Writing - Review & editing. **Vincent Giampietro:** Software, Supervision, Writing - Review & editing. **Katya Rubia:** Conceptualization, Formal anlaysis, Funding acquisition, Investigation, Project administration, Supervision, Writing - Original draft, Writing - Review & editing.

## Declaration of Competing Interest

The authors declare that they have no known competing financial interests or personal relationships that could have appeared to influence the work reported in this paper.
