# Supplementary material for: Neurofunctional and behavioural measures associated with fMRI-neurofeedback learning in adolescents with Attention-Deficit/Hyperactivity Disorder
Source: Neuroimage Clin. 2020 May 26;27:102291. doi: 10.1016/j.nicl.2020.102291 (PMC7287276; doi:10.1016/j.nicl.2020.102291)
Supplement: Supplementary data 1 [file mmc1.docx]

**Supplementary Material**

**1.1 fMRI-NF Data Acquisition and Processing**

Gradient-echo echo planar MR imaging (EPI) and structural data were acquired on a 3T General Electric MR750 scanner with a 12-channel head coil at the Centre for Neuroimaging Sciences, King’s College London. The body coil was used for RF transmission. A T1-weighted structural scan (TR/TE (echo time) = 7.312/3.016 ms, flip angle = 11 degrees, 196 x 1.2 mm slices, matrix size 256 x 256, 27 cm FOV, voxel size = 1.05 x 1.05 x 1.2 mm3), used as structural localizer, was collected at the beginning of each scanning session. fMRI-NF scans were collected using an T2*-weighted gradient echo, EPI sequence (TR/TE = 2000/30 ms, flip angle = 75°, 40 x 3 mm slices with a 0.3 mm slice gap, matrix size 64 x 64, 21.1 cm FOV, voxel size = 3.3 x 3.3 x 3.3 mm^3^). A whole-brain higher resolution gradient echo EPI scan for standard space normalization of individual activation maps was also acquired, aligned to the intercommissural plane with TR/TE = 3.000/30 ms, flip angle = 90°, 43 slices, slice thickness = 3.0 mm, slice gap = 0.3 mm, matrix size 128 x 128, 21.1 cm FOV, voxel size = 1.65 x 1.65 x 3.3 mm^3^.

For the real-time transfer and analysis of the fMRI data, a custom fMRI-NF interface system (Bodurka & Bandettini, 2008) and the Analysis of Functional Neuro Images (AFNI) (Cox, 1996) software were used. The fMRI-NF interface system ran on the scanner hardware to access the fMRI scans as they were reconstructed. These images were then transferred to an external Linux workstation and pre-processed using AFNI, a software package with built-in real-time capacities. Head motion was corrected for in real-time by the AFNI software. The AFNI CA_N27_ML/TT_N template (Eickhoff-Zilles macro labels from Talairach-transformed Colin N27 template) was used to structurally define the target ROIs (ROI_TAR_; rIFC or lPHG) in Talairach space, that were then back-transformed onto each participant’s native brain space before each fMRI-NF session. The ROI included the pars triangularis (14,138 voxels in the Talairach space of the template and 385 voxels when mapped back to fMRI space) and the pars orbitalis (11,484 voxels in the Talairach space of the template and 308 voxels when mapped back to fMRI space). A customised AFNI script automatically created a native-space image mask of the rIFC or lPHG and of the white matter (ROI_REF_; used as reference region to cancel out non-specific global brain effects), based on the T1-weighted structural image that was acquired from session one and a two-volume EPI localizer image that was acquired from the start of each session, matched to the fMRI sequence for the geometric distortion inherent in EPI acquisitions (also used as realignment target for all runs within a visit). The image mask of the pre-selected ROIs was applied to the pre-processed fMRI images and the mean BOLD signal was extracted from each ROI in real-time. Data were time-averaged over 3 time points for smoothing. For each newly acquired brain volume, AFNI calculated a new set of values for each ROI, which were fed to a locally written program, running on another PC, to generate a dynamic visual feedback display by means of the moving rocket. The threshold required for the rocket to ascend was continuously updated based on current performance compared to that of the average of the previous rest block. The following equation was used:

(ROI_TAR_-ROI_REF_)-(ROI_TAR_Previous-ROI_REF_Previous)

where ROI_REF_Previous and ROI_TAR_Previous are the average activation of ROI_REF_ and ROI_TAR_ in the previous rest block. Participants were informed of the NF delay (~6s), caused by haemodynamic delay and data processing time, before each fMRI-NF run.

**1.2 fMRI-NF brain activation analysis for each subject of up to 11 fMRI-NF runs** fMRI data were analysed using version 4.1 of the non-parametric XBAM software package ([www.brainmap.co.uk](http://www.brainmap.co.uk)) (Brammer et al., 1997). XBAM’s non-parametric approach overcomes many issues associated with parametric software packages that lead to higher false positive rates (Bullmore et al., 1999; Eklund, Nichols, & Knutsson, 2016).

Individual and group-level analysis methods are described in detail elsewhere (Alegria et al., 2017; Brammer et al., 1997; Bullmore et al., 1999). In short, fMRI data were realigned to minimise motion-related artefacts and smoothed with a 7.8 mm full-width-at-half-maximum (FWHM) Gaussian filter. A wavelet-based resampling method (Bullmore et al., 2001) was used for the time-series analysis of individual activation. The experimental conditions, NF minus baseline (i.e. the response size of the NF (active block) condition against the baseline-rest block condition), were convolved with 2 Poisson model functions (peaking at 4 and 8s). The weighted sum of convolutions producing the best fit (least-squares) to the time series at each voxel was calculated. A goodness-of-fit statistic (SSQ) was computed at each voxel consisting of the ratio of the sum of squares of deviations from the mean intensity value due to the fitted time series model divided by that of the squares due to the residuals (original minus model time series). The computed SSQ ratio was used in further analyses, where individual maps were then normalised to Talairach space (Talairach & Toumoux, 1988) and a group activation map was produced for each group.

**
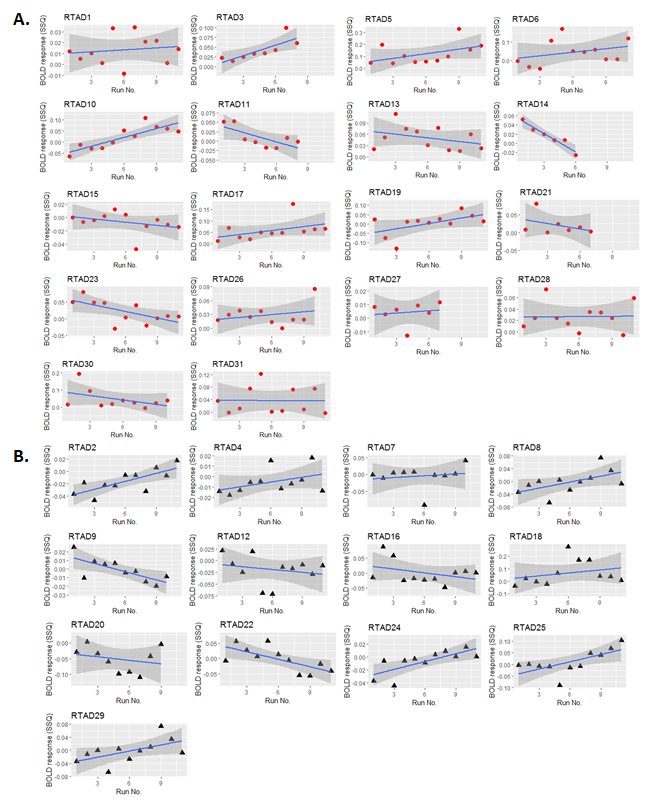
***Figure S1.* Scatterplots of fMRI-NF learning of all participants: A) active rIFC-NF group: scatterplots of mean statistical BOLD activation of rIFC across completed NF runs, B) control lPHG-NF group: scatterplots of mean statistical BOLD activation of lPHG across completed NF runs


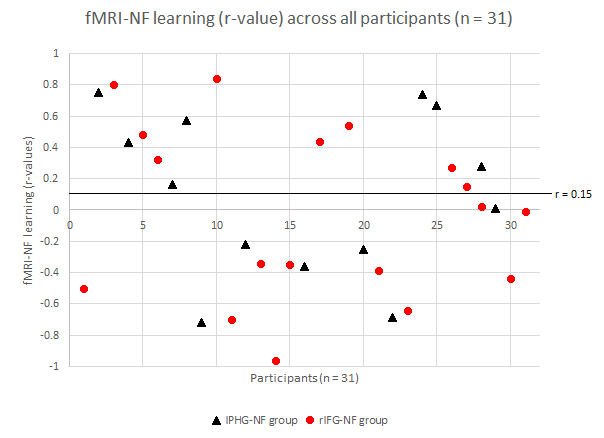


*Figure S2.* Scatterplot of fMRI-NF regulation learning (r-value) across all participants (n =31)

Table S1

*Correlation between baseline primary clinical and neurocognitive measures and the number*

*of completed fMRI-NF runs by participants*

| **Baseline clinical measures** | **Mean (SD)** | **Correlation value r (df = 2, 29)** | **P-value (2-tailed)** |
| --- | --- | --- | --- |
| *ADHD-Rating Scale* |  |  |  |
| Total score | 37.16 (10.13) | -0.015 | 0.937 |
| inattention | 20.29 (4.47) | 0.039 | 0.833 |
| hyperactivity/impulsivity | 16.87 (6.39) | -0.051 | 0.785 |
| *Conner’s Parent Rating Scale* |  |  |  |
| ADHD Index score | 14.81 (4.29) | 0.243 | 0.188 |
| DSM-V inattention | 81.16 (8.53) | 0.091 | 0.627 |
| DSM-V hyperactivity/impulsivity | 85.48 (9.13) | 0.282 | 0.125 |
| **Baseline neurocognitive measures** |  |  |  |
| *Go/No-Go Task* |  |  |  |
| Probability of inhibition (%) | 62.48 (19.02) | 0.023 | 0.904 |
| *Continuous Performance Task (CPT)* |  |  |  |
| Omission errors (%) | 8.29 (6.94) | 0.062 | 0.741 |
| Commission errors (%) | 1.13 (1.43) | -0.048 | 0.797 |
| *Delay Discounting* |  |  |  |
| k median | 0.015 (0.014) | 0.081 | 0.666 |
| *Time Discrimination Task* |  |  |  |
| Total correct | 77.13 (16.63) | 0.180 | 0.332 |
| *Stop Task* |  |  |  |
| Stop signal reaction time (ms) | 116.71 (168.74) | 0.007 | 0.971 |
| *RT combined (CPT, Go-No-Go)* | 369.11 (40.83) | -0.111 | 0.552 |
| *Intra-subject co-efficient of variance combined*  *(CPT, Go-No-Go)* | 0.25 (0.06) | -0.213 | 0.250 |

*Note.* RT combined, combined mean reaction time to targets during Go/No-Go task and Continuous Performance task

Table S2

*Clinical ratings pre- and post-fMRI-NF training for fMRI-NF learners and non-learners*

| 1. **Successful Learners (N = 15)** | **Pre** | **Post** | **Pre-Post** | | |
| --- | --- | --- | --- | --- | --- |
|  | **Mean (SD)** | **Mean (SD)** | **F (1, 14)** | **P-value** | **ES d** |
| *ADHD-Rating scale* |  |  |  |  |  |
| ADHD-RS total score | 37.80 (11.46) | 30.47 (13.25) | 7.50 | **0.016*** | 0.592 |
| ADHD-RS inattention | 20.87 (4.84) | 17.13 (6.69) | 6.46 | **0.023*** | 0.641 |
| ADHD-RS hyperactivity/impulsivity | 16.93 (7.45) | 13.33 (7.36) | 6.22 | **0.026*** | 0.486 |
| *Conner’s Parent Rating Scale (T-score)* |  |  |  |  |  |
| ADHD index | 14.80 (3.93) | 12.24 (6.28) | 3.11 | 0.100 | 0.489 |
| DSM-5 inattention | 80.40 (8.53) | 73.67 (11.45) | 3.43 | 0.085 | 0.667 |
| DSM-5 hyperactivity/impulsivity | 87.00 (7.56) | 81.19 (12.30) | 8.64 | **0.011*** | 0.569 |
| 1. **Non-learners (N = 16)**   *ADHD-Rating scale* |  | | | | |
|  | **Pre** | **Post** | **Pre-Post** | | |
|  | **Mean (SD)** | **Mean (SD)** | **F (1, 15)** | **P-value** | **ES d** |
|  |  | |  |  |  |
| ADHD-RS total score | 36.56 (9.04) | 30.63 (8.27) | 8.433 | **0.011*** | 0.685 |
| ADHD-RS inattention | 19.75 (4.19) | 16.07 (5.44) | 9.713 | **0.007*** | 0.758 |
| ADHD-RS hyperactivity/impulsivity | 16.81 (5.47) | 14.55 (4.49) | 4.009 | 0.064 | 0.452 |
| *Conner’s Parent Rating Scale (T-score)* |  |  |  |  |  |
| ADHD index | 14.81 (4.74) | 10.29 (4.64) | 22.387 | **<0.001*** | 0.964 |
| DSM-5 inattention | 81.88 (8.76) | 71.11 (8.32) | 14.576 | **0.002*** | 1.261 |
| DSM-5 hyperactivity/impulsivity | 84.06 (10.44) | 81.90 (13.70) | 1.825 | 0.197 | 0.177 |
|  |  |  |  |  |  |
|  |  |  |  |  |  |

*Note.* RT combined, combined mean reaction time to targets during Go/No-Go task and

Continuous Performance task; ES d, Cohen’s d effect size.

*Significance level < 0.05

Table S3

*Neurocognitive scores pre- and post-fMRI-NF training for fMRI-NF learners and non-learners*

| 1. **Learners (N = 15)** | **Pre** | **Post** | **Pre-Post** | | |
| --- | --- | --- | --- | --- | --- |
|  | **Mean (SD)** | **Mean (SD)** | **F (1, 14)** | **P-value** | **ES d** |
|  |  |  |  |  |  |
| *Go/No-go Task* |  |  |  |  |  |
| Probability of inhibition (%) | 56.27 (20.87) | 59.83 (20.52) | 0.838 | 0.375 | 0.172 |
| *Time Discrimination Task* |  |  |  |  |  |
| Total correct (%) | 76.67 (19.20) | 73.40 (18.58) | 4.474 | 0.053 | 0.173 |
| *Temporal Discounting Task* |  |  |  |  |  |
| k median | 0.02 (0.02) | 0.02 (0.02) | 0.04 | 0.844 | <0.01 |
| *Continuous Performance Task (CPT)* |  |  |  |  |  |
| Omission errors (%) | 9.93 (8.91) | 8.87 (6.97) | 0.392 | 0.541 | 0.133 |
| Commission errors (%) | 1.33 (1.63) | 0.67 (1.05) | 4.828 | **0.045*** | 0.481 |
| *Stop Task* |  |  |  |  |  |
| Stop Signal Response Time (ms) | 124.93 (202.32) | 152.07 (212.75) | 1.117 | 0.308 | 0.288 |
| *RT combined (CPT, GNG)* | 349.77 (33.76) | 363.39 (35.34) | 2.924 | 0.109 | 0.394 |
| *Intra-subject co-efficient of variance combined (CPT, GNG)* | 0.270 (0.08) | 0.264 (0.06) | 0.137 | 0.716 | 0.085 |
|  |  |  |  |  |  |
| 1. **Non-learners (N = 16)** | **Pre** | **Post** | **Pre-Post** | | |
|  | **Mean (SD)** | **Mean (SD)** | **F (1, 15)** | **P value** | **ES d** |
|  |  |  |  |  |  |
| *Go/No-go Task* |  |  |  |  |  |
| Probability of inhibition (%) | 68.31 (15.56) | 66.92 (19.56) | 0.091 | 0.767 | 0.079 |
| *Time Discrimination Task* |  |  |  |  |  |
| Total correct (%) | 77.56 (14.43) | 77.91 (14.34) | 0.021 | 0.888 | 0.024 |
| *Temporal Discounting Task* |  |  |  |  |  |
| k median | 0.01 (0.01) | 0.02 (0.02) | 3.649 | 0.075 | 0.506 |
| *Continuous Performance Task (CPT)* |  |  |  |  |  |
| Omission errors (%) | 6.75 (4.14) | 6.32 (4.33) | 0.08 | 0.781 | 0.102 |
| Commission errors (%) | 0.94 (1.24) | 0.66 (0.87) | 0.536 | 0.475 | 0.261 |
| *Stop Task* |  |  |  |  |  |
| Stop Signal Response Time (ms) | 109.00 (136.41) | 102.80 (195.82) | 0.051 | 0.825 | 0.036 |
| *RT combined (CPT, Go-No-Go)* | 387.25 (39.31) | 390.56 (38.27) | 0.117 | 0.737 | 0.085 |
| *Intra-subject co-efficient of variance combined (CPT, Go-No-Go)* | 0.237 (0.04) | 0.240 (0.06) | 0.029 | 0.868 | 0.059 |

*Note.* RT combined, combined mean reaction time to targets during Go/No-Go task and Continuous Performance task; ES d, Cohen’s d effect size

*Significance level < 0.05

References

Alegria, A. A., Wulff, M., Brinson, H., Barker, G. J., Norman, L. J., Brandeis, D., . . . Rubia, K. (2017). Real-time fMRI neurofeedback in adolescents with attention deficit hyperactivity disorder. *Human Brain Mapping, 38*(6), 3190-3209. doi:10.1002/hbm.23584

Bodurka, J., & Bandettini, P. (2008). Real-time software for monitoring MRI scanner operation. Proceedings of Human Brain Mapping Conference, Melbourne. *NeuroImage, 41*, S85.

Brammer, M. J., Bullmore, E. T., Simmons, A., Williams, S. C., Grasby, P. M., Howard, R. J., . . . Rabe-Hesketh, S. (1997). Generic brain activation mapping in functional magnetic resonance imaging: a nonparametric approach. *Magnetic Resonance Imaging, 15*(7), 763-770. doi:10.1016/S0730-725X(97)00135-5

Bullmore, E., Long, C., Suckling, J., Fadili, J., Calvert, G., Zelaya, F., . . . Brammer, M. (2001). Colored noise and computational inference in neurophysiological (fMRI) time series analysis: resampling methods in time and wavelet domains. *Human Brain Mapping, 12*(2), 61-78. doi:10.1002/1097-0193(200102)12:2<61::AID-HBM1004>3.0.CO;2-W

Bullmore, E. T., Suckling, J., Overmeyer, S., Rabe-Hesketh, S., Taylor, E., & Brammer, M. J. (1999). Global, voxel, and cluster tests, by theory and permutation, for a difference between two groups of structural MR images of the brain. *IEEE Transactions on Medical Imaging, 18*(1), 32-42. doi:10.1109/42.750253

Cox, R. W. (1996). AFNI: software for analysis and visualization of functional magnetic resonance neuroimages. *Computer and Biomedical Research, 29*(3), 162-173. doi:10.1006/cbmr.1996.0014

Eklund, A., Nichols, T. E., & Knutsson, H. (2016). Cluster failure: Why fMRI inferences for spatial extent have inflated false-positive rates. *Proceedings of the National Academy of Sciences of the USA, 113*(28), 7900-7905. doi:10.1073/pnas.1602413113

Talairach, J., & Toumoux, P. (1988). *Co-planar sterotaxic atlas of the human brain: 3-dimensional proportional system*. Stuttgart: Thieme.
